# An Easy Method of Destroying Tsetse Flies

**Published:** 1910-07-02

**Authors:** 


### AN EASY METHOD OF DESTROYING TSETSE FLIES.

Mr. Maldonado, manager of one of the estates on the Island of Principe, has devised a method of destroying *Glossina palpalis*. Noticing that the flies attacked the backs of the labourers when they were occupied in mowing grass and were consequently in a stooping posture, he ordered that such labourers should wear, while in the woods, a black cloth covering their backs, coated with a glutinous substance on its outer surface. While the Portuguese Commission was in the island there were four persons who went about with these cloths. The Commission often asked Mr. Maldonado to send men with black cloths to places where they had seen a large number of flies. As a rule two men were enough in the short space of a week to make these places passable. On the first days the numbers taken would be 1,500 to 2,000; at the end of the week fifteen or twenty. The method has been tried on two other estates with the same favourable result.

